# Recombinase Polymerase Amplification Assay—A Simple, Fast and Cost-Effective Alternative to Real Time PCR for Specific Detection of *Feline Herpesvirus-1*

**DOI:** 10.1371/journal.pone.0166903

**Published:** 2017-01-03

**Authors:** Jianchang Wang, Libing Liu, Jinfeng Wang, Xiaoxia Sun, Wanzhe Yuan

**Affiliations:** 1 Inspection and Quarantine Technical Center of Hebei Entry-Exit Inspection and Quarantine Bureau, Xinhua District, Shijiazhuang, Hebei, China; 2 College of Veterinary Medicine, Agricultural University of Hebei, Baoding, Hebei, China; Oklahoma State University, UNITED STATES

## Abstract

*Feline herpesvirus 1* (FHV-1), an enveloped dsDNA virus, is one of the major pathogens of feline upper respiratory tract disease (URTD) and ocular disease. Currently, polymerase chain reaction (PCR) remains the gold standard diagnostic tool for FHV-1 infection but is relatively expensive, requires well-equipped laboratories and is not suitable for field tests. Recombinase polymerase amplification (RPA), an isothermal gene amplification technology, has been explored for the molecular diagnosis of infectious diseases. In this study, an exo-RPA assay for FHV-1 detection was developed and validated. Primers targeting specifically the thymidine kinase (TK) gene of FHV-1 were designed. The RPA reaction was performed successfully at 39°C and the results were obtained within 20 min. Using different copy numbers of recombinant plasmid DNA that contains the TK gene as template, we showed the detection limit of exo-RPA was 10^2^ copies DNA/reaction, the same as that of real time PCR. The exo-RPA assay did not cross-detect *feline panleukopenia virus*, *feline calicivirus*, *bovine herpesvirus-1*, *pseudorabies virus* or *chlamydia psittaci*, a panel of pathogens important in feline URTD or other viruses in Alphaherpesvirinae, demonstrating high specificity. The assay was validated by testing 120 nasal and ocular conjunctival swabs of cats, and the results were compared with those obtained with real-time PCR. Both assays provided the same testing results in the clinical samples. Compared with real time PCR, the exo-RPA assay uses less-complex equipment that is portable and the reaction is completed much faster. Additionally, commercial RPA reagents in vacuum-sealed pouches can tolerate temperatures up to room temperature for days without loss of activity, suitable for shipment and storage for field tests. Taken together, the exo-RPA assay is a simple, fast and cost-effective alternative to real time PCR, suitable for use in less advanced laboratories and for field detection of FHV-1 infection.

## Introduction

*Feline herpesvirus 1* (FHV-1), an enveloped double-stranded DNA virus, belongs to the Varicellovirus genus of the subfamily Alphaherpesvirinae [1]. It is the major pathogen of feline upper respiratory tract disease (URTD) and ocular disease. It is estimated that FHV-1 infection accounts for approximately 50–75% of URTD in susceptible cats and more than 90% of the cats are seropositive to FHV-1 [2, 3]. So far, only one serotype of FHV-1 has been identified but its distribution is worldwide [1]. Like other alpha herpesviruses, FHV-1 induces latency in the trigeminal ganglions after the primary infection and the latent virus can be activated by stress and other factors that compromise host immune function [1, 4]. About 45% of latently infected cats shed virus periodically [5]

Different diagnostic methods for FHV-1 infection have been described. Virus isolation can be applied for the diagnosis of acute FHV-1 infection. However, it often yields false negative results for FHV-1 detection in latent infection. As over 90% of cats are seropositive to FHV-1, and seroprevalence does not vary significantly among clinically normal and diseased cats, it is believed that antibody testing hardly aids the diagnosis of FHV-1 infection [3]. Polymerase chain reaction (PCR) remains the gold standard diagnostic tool for FHV-1 infection with real time PCR having higher sensitivity than conventional PCR [2, 6–8].

Recombinase polymerase amplification (RPA), an isothermal nucleic acid amplification technology [9], has been explored for molecular detection of diverse pathogens [10–13]. The RPA process employs three enzymes-a recombinase, a single-stranded DNA-binding protein (SSB) and a strand-displacing polymerase. The recombinase is capable of pairing oligonucleotide primers with homologous sequence in the target DNA. SSB then binds to the displaced strand of DNA and prevents the dissociation of primers. Finally, the strand displacing polymerase begins DNA synthesis where the primer has bound to the target DNA. With the use of two opposing primers, exponential amplification of the target sequence with RPA can be achieved at a constant temperature in 10–20 min. The RPA product can be measured in real-time using different probes with fluorescence detection device. In this study, using the fluorescent TwistAmp^®^ exo Probe (TwistDX, Cambridge, UK), we developed and validated a real-time RPA assay for rapid and specific detection of FHV-1.

## Materials and Methods

### Virus strains and *chlamydia psittaci*

*Feline herpesvirus-1* (FHV-1), *feline panleukopenia virus* (FPV) and *feline calicivirus* (FCV) were purchased from ATCC (ATCC, Manassas, USA) and propagated in feline kidney F81 cells (Tiandz Inc., Beijing, China). *Bovine herpesvirus-1* (BHV-1) was isolated in our laboratory and the identity was validated by aligning the glycoprotein B gene sequence with reference sequences (GenBank: JX898220, JN787952 and AJ004801). *Pseudorabies virus* (PRV, Barth-K61 strain) was obtained from the Ringpu Bio-technology Inc. (Baoding, China) and propagated in porcine kidney PK-15 cells (ATCC). *Chlamydia psittaci* (*C*. *psittaci*) was kindly provided by Dr. Cheng He, China Agricultural University, Beijing, China [14], and propagated in murine McCoy fibroblast cells (ATCC).

### Nucleic acid extraction and reverse transcription

Viral nucleic acid was isolated from supernatant collected from virus infected cell culture using the TIANamp DNA Extraction Kit (Tiangen Biotech Co. Ltd., Beijing, China) (for viral DNA) or Trizol Reagent (Invitrogen, Beijing, China) (for viral RNA) according to the manufacturers’ instructions. *C*. *psittaci* nucleic acid was extracted from *C*. *psittaci* infected McCoy fibroblast cell lysate using the TIANamp DNA Extraction Kit. Nucleic acid was quantified using a ND-1000 spectrophotometer (NanoDrop, Wilmington, USA). One μg of FCV RNA was reverse transcribed using the Primescript II 1st strand cDNA Synthesis Kit (Takara Bio Inc., Dalian, China) according to the manufacturer’s instructions. The synthesized cDNA was purified using the cDNA Purification Kit (Takara Bio Inc.) and quantified using a ND-1000 spectrophotometer. All DNA and cDNA templates were stored at -20°C until assays were performed.

### Construction of recombinant plasmid carrying FHV-1 thymidine kinase (TK) gene

FHV-1 TK gene was amplified by PCR using following primers: forward primer, 5’- GGACAGCATAAAAGCGATTG-3’; and reverse primer, 5’-CAACTAGATTTCCACCAGGA-3’. The template was FHV-1 nucleic acid. The TK gene was cloned into plasmid pMD19-T using the pMD19-T Vector Cloning Kit (Takara Bio Inc.) following the manufacturer’s instructions. Subsequently, recombinant clones were screened by PCR and validated by DNA sequencing. The resultant recombinant plasmid, termed pMD19-T-TK, was replicated in *E*.*coli* DH5α cells, extracted and purified using the SanPrep Plasmid MiniPrep Kit (Sangon Biotech Co. Ltd, Shanghai, China). Plasmid DNA was quantified using a ND-1000 spectrophotometer.

### Exo-RPA primers and the probe

To date, specific programs for RPA primer design are not available. For this study, primers for the RPA assay were designed using the Primer 3.0 program following the “primer design considerations” for RPA (http://www.twistdx.co.uk/images/uploads/docs/Appendix.pdf). According to the reference sequence of FHV-1 (GenBank: FJ478159), 3 pairs of primers targeting the conserved region of the TK gene were designed. After trials, 1 pair of primers was chosen for this study. Sequences of the primers and the probe were as follows: forward primer, 5’-GAGTTTAACGGCGAAGTACCTGGTCAGAGCG-3’; reverse primer, 5’-CACCACGTCGTTTCCGGTCCTGGACGGCATA-3’; and the probe, 5’-CCAGAACCAATGCTATACTGGCGTAGTCTC[FAM-dT]-THF-[BHQ-dT]GAAACTGATGTTGTCGGTGG-3’. Primers and the probe were synthesized by Takara Bio Inc.

### Exo-RPA reactions

**Fig 1:**
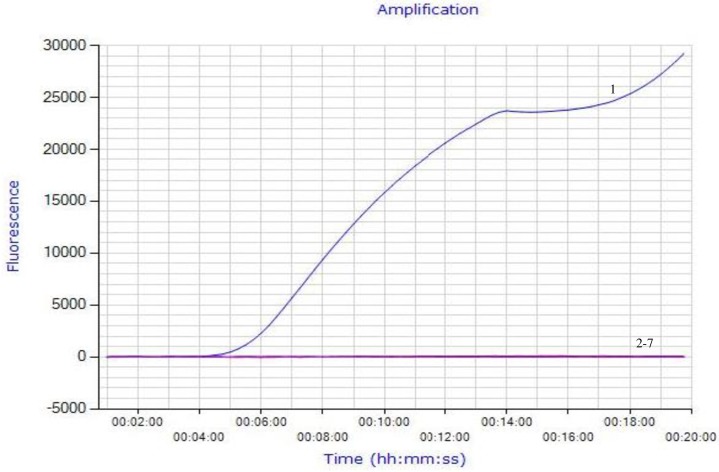
Specificity analysis of the exo-RPA assay. As shown in this figure, only FHV-1 DNA was positively amplified (curve 1). In contrast, *feline calicivirus*, *chlamydia psittaci* and other viruses commonly seen in cats, i.e., FPV, BHV-1, PRV, were all tested negative (curves 2–6, respectively). Curve 7 used nuclease-free water as a negative control. Shown in this figure is one representative plot out of 5 independent reactions.

**Fig 2:**
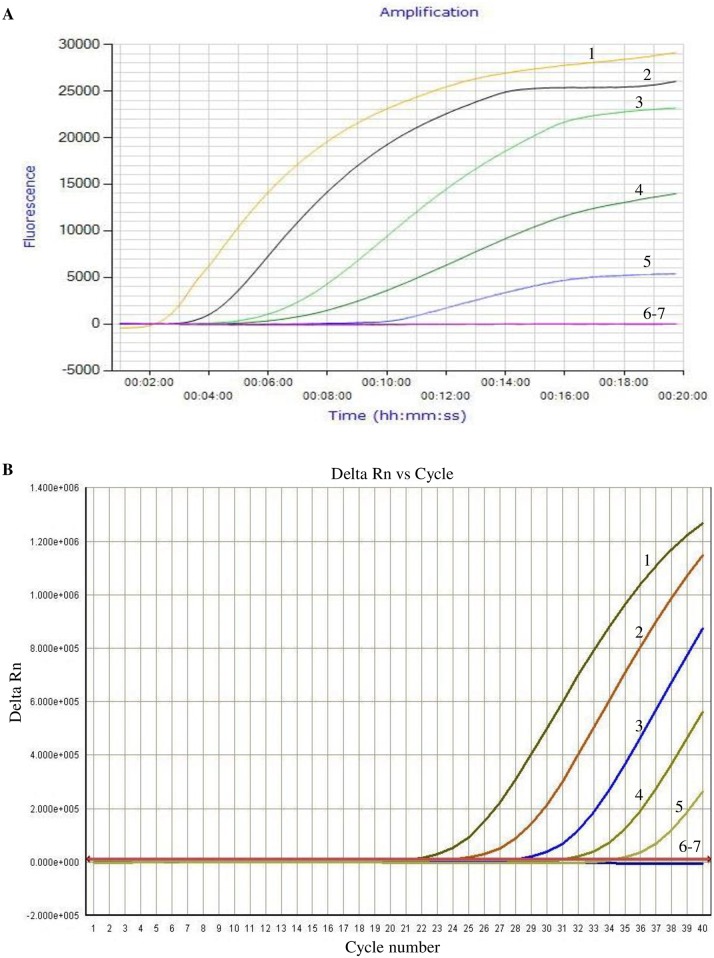
Sensitivity analysis of the exo-RPA assay. Different copy numbers of plasmid pMD19-T-TK DNA (10^6^ to 10^0^ copies) were amplified by either RPA reactions or real time PCR. As shown in this figure, the detection limit was 10^2^ copies of DNA/reaction for both the exo-RPA assay (panel A) and real time PCR (panel B). The copy numbers used as template for curve 1–7 were 10^6^, 10^5^, 10^4^, 10^3^, 10^2^, 10^1^ and 10^0^, respectively. Shown in this figure is one representative plot out of 5 independent reactions for RPA and real time PCR, respectively.

The exo-RPA reaction was performed in a 50 μL volume using a TwistAmp^™^ exo kit (TwistDX, Cambridge, UK). Other components included 420 nM each RPA primer, 120 nM exo probe, 14 mM magnesium acetate, and 1 μL of nucleic acid. All reagents except for the template and magnesium acetate were prepared in a master mix, which was distributed into each 0.2 ml reaction tube containing a dried enzyme pellet. One μL of nucleic acid template was added to the tubes. Subsequently, magnesium acetate was pipetted into the tube lids, the lids were closed carefully, and magnesium acetate was centrifuged into the rehydrated material using a minispin centrifuge. The sample was vortexed briefly and spun down. The tubes were immediately placed in the Genie III Instrument (OptiGene Limited, West Sussex, UK) and incubated at 39°C for 20 min. The fluorescence signal was monitored in real-time. A cutoff value of 3000 (fluorescence signal at the Y-axis, see Figs 1 and 2A) after 20 min of amplification was established to distinguish positive from negative results.

### Specificity and sensitivity analysis

Specificity of the RPA assay was evaluated using 10 ng of pathogen genomic DNA or cDNA as template. A panel of pathogens considered important in cats, i.e., FPV, FCV, PRV, BHV-1 and *C*. *psittaci* were tested with the RPA assay.

To evaluate the sensitivity of the RPA assay, plasmid pMD19-T-TK DNA was 10-fold serially diluted to achieve DNA concentrations ranging from 10^6^ to 10^0^ copies/μL and 1 μL of each dilution was then amplified by RPA reactions. Real time PCR was also performed using 1 μL of each dilution as the template. Real time PCR primers and the probe were designed according to the published sequence (GenBank: FJ478159) as follows: forward primer, 5’-GGACAGCATAAAAGCGATTG-3’; reverse primer, 5’-AACGTGAACAACGACGCAG-3’; and the probe, 5’-FAM-AATTCCAGCCCGGAGCCTCAAT-BHQ1-3’. Primers and the probe were synthesized by Takara Bio Inc. Real time PCR was performed using the Premix Ex Taq Master Mix (Takara Bio Inc.) according to the manufacturer’s instruction. The reaction was completed on the ABI 7500 Real-Time PCR System in following steps, 95°C for 3 min and then 40 cycles of 95°C for 15 s and 60°C for 30 s. The fluorescent dye FAM was used as a reporter dye in real time PCR. The dye can incorporate into the amplified product, and the fluorescent intensity is proportional with the relative amount of the PCR product, which can be measured in real time and is reflected by the Y-axis value, so called delta Rn value, on a real time PCR plot. The Rn value stands for normalized reporter value. The delta Rn value is the Rn value of an experimental reaction minus the Rn value of the baseline signal generated by the instrument. A cutoff Delta Rn value of 2.000e + 005 (see Fig 2B) after 40 cycles of amplification was established to distinguish positive from negative results. 2.000e + 005 is a notation for 2 x 10^5^. The "N+m" formula, where n and m are numbers, is commonly used for a very high number. The format "2.000e + 005" was generated automatically by the PCR machine. The sensitivity between the RPA reaction and real time PCR was compared. The results from 5 independent reactions using the lowest detectable copy number of the template (10^2^) were statistically analyzed and the co-efficient of variations was calculated to determine the reproducibility of the assay.

### Validation with clinical samples

From 2013 to 2015, 120 clinical samples, i.e., nasal and ocular conjunctival swabs, were collected from cats with conjunctivitis, rhinitis or symptoms of upper respiratory tract infections and sent to our laboratory for test. They were from different veterinary clinics and two breeding catteries in Hebei Province, China. The swab was placed in 200 μL sterile phosphate-buffered saline. After centrifugation at 1000×g for 10 min at 4°C, the supernatant was collected and applied for viral DNA extraction using the TIANamp DNA Extraction Kit. DNA from each sample was finally eluted in 20 μL of nuclease free water and stored at a -80°C freezer until assays were performed (1 μL DNA preparation was assayed by both RPA and real time PCR).

## Results and Discussion

The most important etiologic agents for URTD that are prevalent in cats include FHV-1, *feline calicivirus* and Chlamydia [1, 15–17]. Identification of the particular agents in URTD is essential for a better understanding of the pathogenesis of individual infections and corresponding clinical management. RPA assays have been explored for molecular diagnosis of diverse pathogens [10–13]. In this study, an exo-RPA assay was developed for rapid and specific detection of FHV-1. Specificity analysis showed that only FHV-1 DNA was positively amplified (Fig 1). In contrast, *feline calicivirus*, *Chlamydia psittaci* and other viruses commonly seen in cats, i.e., FPV, BHV-1, PRV, were all tested negative (Fig 1). These results were reproducible in 5 independent reactions, demonstrating high specificity of the exo-RPA assay for FHV-1 detection.

Different copy numbers of pMD19-T-TK plasmid DNA were assayed by exo-RPA and real-time PCR. As shown in Fig 2A, the detection limit of the exo-RPA method was 10^2^ copies/reaction. Real-time PCR also had a detection limit of 10^2^ copies/reaction (Fig 2B). Thus, in terms of assay sensitivity, these data suggest that exo-RPA is comparable to real time PCR. The co-efficient of variations from 5 independent reactions (10^2^ copies of template) was demonstrated in Table 1, showing high reproducibility for both real time PCR and the RPA assay. A total of 120 clinical samples were tested by exo-RPA and real-time PCR. The results showed a complete diagnostic agreement between RPA and real time PCR (47 positive and 73 negative as shown by both methods).

**Table 1:** The co-efficient of variation from 5 independent reactions.

Amount of template (copies)	RPA			Real time PCR		
	Mean	SD	CV	Mean	SD	CV
10^2^	14.10	0.38	2.72%	38.88	0.53	1.35%

Mean number for RPA is the average of the threshold time (min) from 5 independent RPA reactions; Mean number for real time PCR is the average of the Ct numbers from 5 independent reactions. SD: standard deviation; CV: co-efficient of variation.

Reubel et al described a conventional PCR assay for FHV-1 detection [18]. They used agarose gel electrophoresis together with ethidium bromide staining to visualize the PCR product and observed that as low as 3x10^3^ copies of FHV-1 genomic DNA could be detected. Weigler et al developed a real time PCR method for FHV-1 detection, which had a detection limit of 240 copies of FHV-1 DNA [7], in line with our results obtained with real time PCR and the RPA assay. Compared with real time PCR, RPA has several advantages. First, RPA reactions are done at a constant temperature, requiring less-complex instrument. Secondly, RPA reactions are usually completed in 10–20 min, much faster than real time PCR that often takes > 60 min. Thirdly, commercial RPA reagents in vacuum-sealed pouches can tolerate temperatures up to room temperature for days without loss of activity (http://www.twistdx.co.uk/images/uploads/docs/TA01cmanual_Combined_Manual_RevI.pdf), eliminating the need of complex shipment and storage containers, which is convenient and fitting for field performance of rapid point-of-care diagnostic tests. Lastly, it has been shown that RPA primers and probes carrying 5–9 base pair mismatches do not influence the performance of RPA reactions [13, 19], while such base pair mismatches can cause real-time PCR small probes to fail [20].

With the development of portable devices specifically designed for out-door use, e.g., Genie III Instrument, which are light and incorporate a rechargeable battery that can support operation for a complete day, the RPA assay developed in this study could become a useful tool for field diagnostic tests of FHV-1 infection. To our knowledge, this is the first report on the development of an RPA assay for the detection of FHV-1. In conclusion, the RPA assay is a simple, fast and cost-effective alternative to real time PCR for specific detection of FHV-1.
